# High Expression of Sphingosine Kinase 1 in Estrogen and Progesterone Receptors-Negative Breast Cancer

**Published:** 2017-05-30

**Authors:** Azadeh-Sadat Nazouri, Ommolbanin Asadpour, Shahriar Dabiri, Bahram Pourseyedi, Mohamad Reaza Lashkarizadeh, Hamid Zianalinejad

**Affiliations:** 1 *Dept. of Biology, Science Faculty, Shahid Beheshti University, Tehran, Iran*; 2 *Pathology and Stem Cell Research Center, Pathology Department, Afzalipour School of Medicine, Kerman University of Medical Sciences, Kerman, Iran*; 3 *Dept. of Medical Biotechnology, Faculty of Medical Sciences, Tarbiat Modares University, Tehran, Iran*; 4 *Surgery Department, Kerman University of Medical Sciences, Kerman, Iran*

**Keywords:** Sphingosine Kinase, Phosphate, Estrogen and Progesterone, Receptors, Real–time PCR, Breast Cancer

## Abstract

**Background & objective:**

Breast cancer is the leading cause of cancer related death in females. Sphingosine kinase 1 (SPHK1) and its product sphingosine-1-phosphate (S1P) are the essential key regulator molecules in breast cancer through their ability to promote cell proliferation, angiogenesis, cell proliferation, and lymphagiogenesis. *SPHK1* is overexpressed in multiple types of cancer including breast cancer and is associated with resistance to treatment. The current study aimed at investigating the expression of *SPHK1* in estrogen and progesterone receptors (ER, PR) negative in comparison to ER, and PR positive breast cancer and their normal controls, and also finding the relationship between *SPHK1 *expression and high body index (BMI) in the selected groups with breast cancer.

**Methods:**

A total of 120 human breast cancer tissue specimens were analyzed for *SPHK1* expression using Quantitative Real–Time Polymerase Chain Reaction (q RT-PCR) assay. Detection of hormonal status of breast cancer tissue samples was conducted by immunohistochemical assay.

**Result:**

The current study findings showed that the level of *SPHK1*expression in the breast cancer tissue was significantly higher in patients with estrogen and progesterone negative receptors, compared to the ones without them (P-value< 0.05). The obtained data confirmed that the obesity in patients with ER negative was higher than the ones with positive receptors (BMI> 25).

**Conclusion:**

The current study showed that expression of *SPHK1*gene was higher in the patients with ER and PR negative breast cancer and high BMI, compared with other groups.

## Introduction

Breast cancer is one of the most common cancers and is the leading cause of cancer–related death among females worldwide including Iran (1); 15% to 20% of the risk factors of developing this cancer 

is associated with genetics(2). The prevalence of breast cancer in Iran is among the females in the age range of 15 to 84 years; the age range of the participantswas40 to 49 years. In the Iranian population, 77% and less than 5% of breast cancers are infiltrative ductal and lobular carcinoma, respectively (3). Different parameters such as tumor size, lymph node (LN) status, histological type and grade, status of hormonal receptors(ER and PR), and Her-2/ neu status are used for the prognosis of breast cancer.ER and PR expression is observed in more than 70% of breast cancer cases. Studies determined that the expression of ER and PR is associated with better response to hormone therapy (4, 5). As such patients lack estrogen and progesterone receptors, they are resistant to treatment that target these receptors and associated with poor prognosis (6). Therefore, finding new molecules, signaling pathways, and development of new therapies are important goals to treat breast cancer.Sphingosine-1-phosphate (S1P), ceramide, and sphingosine known as sphingolipid metabolites play different roles in molecular signaling of essential biological processes and the balance between them determines the cell fate; it means that when the balance goes towards ceramide and sphingosine, a cell is selected for death pathways and if the levels of S1P increase, a cell goes toward the survival and proliferation pathways. Hence, it is suggested that the perfect setting of these metabolites can affect the treatment of cancer (7, 8).The balance is set by several regulators. Sphingosine kinases (SphKs) are introduced as unique regulators that produce S1P and decrease other metabolites (9).Anti-apoptotic lipid, S1P, is synthesized by SphKs, especially Sphingosine kinase 1 (SPHK1).Two distinct is of orms are known for SphKs; SPHK1 that regulates the essential processes of cancer progression, and SPHK2 that is much less known about its biological actions in cancer. High expression of SPHK1 is detected in multiple types of cancers, which can be associated with tumor angiogenesis and resistance to radiation and chemotherapy(10).SPHK1,as a cytosolic enzyme, is stimulated by binding multiple growth factors through their tyrosine kinase receptors (RTKs) to produce higher S1P (10, 11). Therefore, S1P function, both as an intracellular second messenger (12) and as specific ligand, activates G protein–coupled receptors (GPCRs) (9), and their downstream signaling to induce transactivation of various RTKs (10). SPHK1 has an important role in activation of cell proliferation, inflammatory response, migration and dysfunction in apoptosis, and its expression in tumor cells can lead to tumor growth, drug resistant, oncogenic transformation, neovascularization of tumors, and metastatic spread (13). SPHK1 was found from rat renal cells for the first time. It is located on chromosome 17 q25.2 and has eight exons. Its expression was shown in multiple tissues such as brain, heart, lungs, spleen, and hematopoietic immune system (14). Evidence determined that *SPHK1* has oncogenic manner, which highlighted it as oncogenic enzyme (15), and can be a chemotherapeutic target because of its oncogenic characteristic and high expression of this gene and its product, S1P depends on many growth factors, which leads this gene and S1P toward stimulatory effects on tumor angiogenesis and cell motility that are critical for metastasis (10). It is overexpressed in many cancers such as lungs, colon, prostate (16), acute leukemia (17), clear cell renal carcinoma (18), and ovarian cancer (19). Overexpression of *SPHK1* required to transform NIH3T3 fibroblasts to tumor phenotype in nude mice (15); and also the upregulation of this enzyme is associated with erythroleukemia (20). Inhibition of this gene reduces cell growth in triple negative breast cancer and clear cell renal carcinoma (ccRCC) (18), and decreases LN metastasis and tumor size (21). The current study aimed at investigating whether *SPHK1 *is expressed highly in ER and PR negative compared to ER and PR positive breast cancers and their normal tissue, and also the relationship of this expression with BMI in patients with breast cancer.

## Materials and Methods

The current case-control study selected 120 formalin fixed, paraffin embedded (FFPE) samples of patients aged 30 to 70 years from pathology wards of hospitals in Kerman, Iran, from 2010 to 2015.To calculate BMI, patients’ demographic were recorded. Samples were collected after the approval of study protocol by the local ethical committee, and all cases signed the informed consent.

The 120 samples were divided into four groups of 30. Tumor groups included ER negative and ER positive breast cancer and the control groups including the normal tissue of selected tumor specimens. Immunohistochemistry (IHC) staining for ER and PR was performed by Dako Kit (Denmark).

**RNA extraction from FFPE tissue**

Three sections of 10-uM thick of each paraffin block were cut with microtome to study mRNA gene expression. Excess paraffin was trimmed and, then, total RNA was extracted from sectioned tissue blocks, using the absolutely RNeasy FFPE kit (QIAGEN, Germany). The integrity and size distribution of total RNAs were checked by 2% agarose gel electrophoresis with SYBR safe™, and two distinct ribosomal RNA bands were observed in each sample. The purity of RNAs was estimated by UV–spectrophotometry (Thermo Scientific, USA) in 10 uMTris-Cl buffer (pH=7.5). TheA260/280 ratio was 1.9 to 2.1.

**Reverse transcription**

Total RNAs extracted from FFPE tissue sections were reverse- transcripted in a final volume of 20 uL by the first-strand cDNA synthesis kit(Thermo, USA). To detect and exclude any interference by residual DNA contamination, polymerase chain reaction (PCR0 was performed with the same total RNA samples without reverse transcriptase. Products were electrophoresedon 2% agarose gel.

**Real – time quantitative reverse transcription PCR**

**Figure 1:**
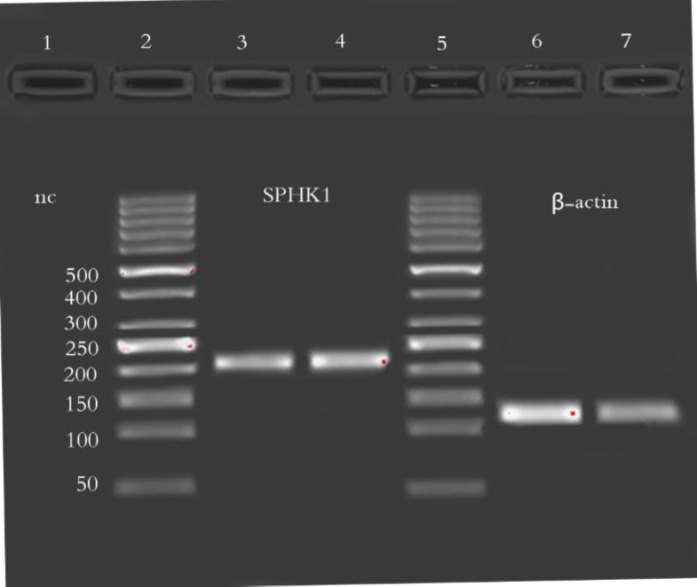
Real-time PCR products of SPHK1 and β–actin electrophoresedon 2% agarose gel in 80 constant voltage; from left to right 1: negative control;2, 5: 50 bp ladder;3, 4: SPHK1 band(204 bp);6,7: β–actin band (120 bp)

Real–time quantitative RT-PCR amplification for SPHK1 and β-actin mRANs were performed using the 7500 real time PCR system (Applied Biosystem- USA),power SYBR Green PCR master mixes (Real Q plus 2x master Mix Green, AMPLIQON, Denmark), and they were run in duplicate 20 uL reactions using 50 ng of synthesized cDNA per reaction well, final concentration of primers was5 pM. PCR cycling program was as follows: 95°C for 15 minutes for one cycle, 95°C for 20 seconds, 62.4°C for 30 seconds, and 72°C for 30 seconds for 45 cycles. To ensure that the resulting signals were derived from extracted RNAs, PCR also was performed fora similar control, but without the RT reaction (Figure 1). 

REST384 version 2 (2006) software was used to obtain data and compare mean values among the groups.

**Primer Design**

According to Intron–spanning of SPHK1 gene, a pair of primers was designed by Perl-primer (v.1.1.19) software. To determine the quality of synthesized cDNA and normalization of PCR reaction;β-actin, as a house keeping gene, was used and previously designed (NCBI reference sequence NM 001101.3) (Table 1).

**Table I:** Information of Designed Primers

Amplicon Size (bp)	Tm(°C)	Primer Sequence (5′-3′)	Gene
**204**	62.4	F: CTTCCTTGAACCATTATGCTG R:GCCGATACTTCTCACTCTC	*SPHK1*
**120**	62.4	F: ACCACCTTCAACTCCATCATG R: CTCCTTCTGCATCCTGTCG	β -actin

Abbreviations: F, forward primer, R, reverse primer, Tm, annealing temperature; and bp, base pair

## Results

**Figure 2:**
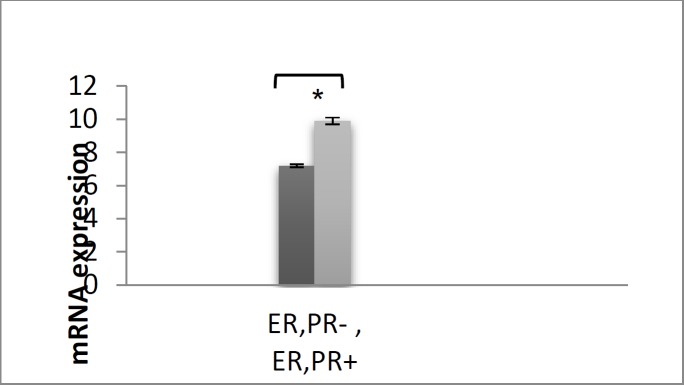
*SPHK1*and *β–actin *expression levels in the studied groups; *P*-value <0.05

To compare the expression level of SPHK1 in ER and PR positive and negative Iranian breast cancer, and with their normal breast tissue, quantitative RT – PCR was performed. All RT-PCR reactions were repeated twice to minimize the experimental error. REST384- version 2 (2006) software was employed for data analysis. The current study data suggested a significant difference between SPHK1 expression level in negative receptor of estrogen and progesterone, compared with the positive receptors of breast cancer (P-value=0.0177) (Figure 2). 

**Figure 3:**
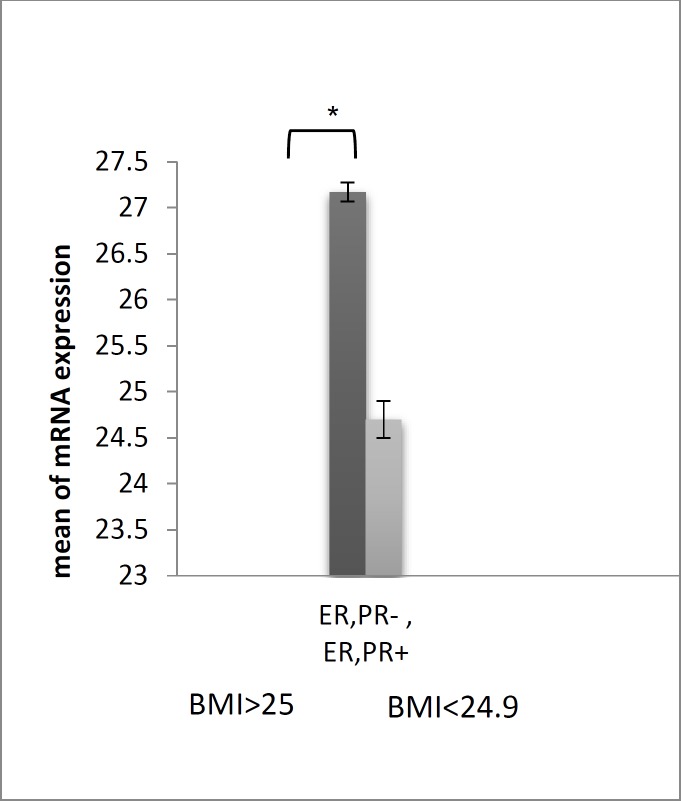
The mean weight of ER and PR negative receptors in comparison with that of ER and PR positive receptors breast cancer in the studied groups; P-value <0.05

Comparison of BMI between the two tumor groups demonstrated that patients with negative receptors had higher BMI (> 25) than the patients with positive receptors (Figure 3).

The current study confirmed no statistically significant relationship between the expression level of SPHK1 in negative and positive normal breast tissue, compared to their tumor samples (P-values= 0.641 and 0.48). 

## Discussion

Normal breast stroma tissue is changed into active stroma, during tumorigenesis. Stromal microenvironment of tumor cells is reported as a new factor in cancer progression. The metastatic rate of multiple cancers, including breast cancer, depends on the bilateral communication between tumor cells and their surrounding microenvironment (22, 23, 24). SPHK1 and its product (S1P), known as biolipid microenvironment, have critical roles in regulation of cell proliferation, differentiation, and angiogenesis (10, 25). The level of SPHK1 and its product S1P is elevated in multiple cancers, including breast cancer, especially in ER and PR negative tumors (26). In this regard, a study in 2000 observed that *SPHK1* displayed higher expression among ER negative breast cancer (27). In agreement with that, Longs et al. and Pyne et al. reported that expression of *SPHK1*hadno effective impact, and its inhibition might be of little use in ER- positive breast cancer treatment (28,29), while high expression of *SPHK1*is associated with shorter disease-specific survival in ER negative tumors (30). Also, further confirming results showed that *SPHK1* expression increased up to four-fold in breast cancer, compared with the control groups (31).The current study confirmed the previous studies and showed a strong positive correlation between *SPHK1 *expression in ER and PR negative tumors in comparison with ER and PR positive breast cancer. Although the mechanisms of more highly *SPHK1* expression in negative receptors of breast tumor is unclear, it can be due to its higher proliferation activity (27), which is consistent with the anti-apoptotic effect of S1P (10). Similarity of the oncogenic lipid kinase (SPHK1) to leptin has led many studies done on the relationship between this gene, obesity, and breast cancer; although these mechanisms and pathways were linked with poor prognosis (32). Blachino et al., showed that fat tissue of obese females has high content of SPHK1, which induces proliferative response, offering that obesity maybe a factor for SPHK1 levels (33). More studies suggested that expression of *SPHK1*and leptin receptor (LEPR) elevated in ER negative breast tumor with higher BMI (34, 35). Further investigations showed that leptin, as a prominent adipokine, has an essential role in breast tumor prognosis, advanced stage, and metastasis (36, 37,38). A new pathway suggested that leptin induces phosphorylation of STAT3 and SFK, and finally increases*SPHK1* mRNA and metastasis in ER negative breast cancer (32). Similarly, the current study results indicated that in ER negative breast cancer, *SPHK1 *is expressed more highly associated with the elevation of BMI. IHC assay of breast cancer showed that the major source of *SPHK1 *expression is carcinoma cells (10) that finally lead to produce S1P, which is the cause of angiogenesis (39), lymphangiogenesis (40), cell survival, and migration (41). As the expression of SPHK1 is associated with resistance to radiation and chemotherapies (42, 43), using *SPHK1 *inhibitors and its specific analogs can be effective in the treatment of ER negative breast tumors. In this regard several studies in cancer cell lines and animal models were conducted (21, 44).

## Conclusion

 Overall, the results of the current study showed that *SPHK1* was expressed more highly in breast tumors, especially in negative receptors, and its expression can be associated with higher BMI in the patients; although further investigations are needed to find out its mechanisms as well as its subsequent influence on breast tumor.
